# Anisotropy governs strain stiffening in nanotwinned-materials

**DOI:** 10.1038/s41467-018-03972-9

**Published:** 2018-04-23

**Authors:** Seyedeh Mohadeseh Taheri Mousavi, Guijin Zou, Haofei Zhou, Huajian Gao

**Affiliations:** School of Engineering, Brown University, Providence, RI 02912 USA

In their papers, Li et al.^1,2^ proposed an indentation strain stiffening mechanism to explain the experimentally reported ultra-high hardness of nanotwinned (nt-) cBN^3^ and nt-diamond^4^ at extremely small twin boundary (TB) thicknesses (*λ* ≤ 5 nm). Here, however, we show that the strain stiffening mechanism proposed by these authors is not exclusive to nt-covalent-bonding materials and also exists in nt-metals which are known to exhibit a softening behavior below a critical twin spacing^5,6^.

To demonstrate this, molecular dynamics (MD) simulations were first conducted on a single crystalline Cu sample with dimensions of 40 × 20 × 56 nm^3^. Periodic boundary conditions were imposed at the boundaries of the sample. The embedded atom method potential for Cu was adopted to describe the interatomic interactions^7^, and the Nose–Hoover thermostat was used to maintain an NPT ensemble. The time step is set at 1 fs. The sample was relaxed at 1 K for 200 ps. A total shear strain of 90% was applied along the (111)$[\bar 1\bar 12]$ (hard shearing) and (111)$[11\bar 2]$ (weak shearing) directions at a constant strain rate of 10^9^ s^−1^. The simulated stress-strain responses displayed in the left inset of Fig. 1 shows that continuous loading along the weak direction causes all atomic layers to be transformed into the hard direction, and the sample exhibits strain stiffening.

**Fig. 1:**
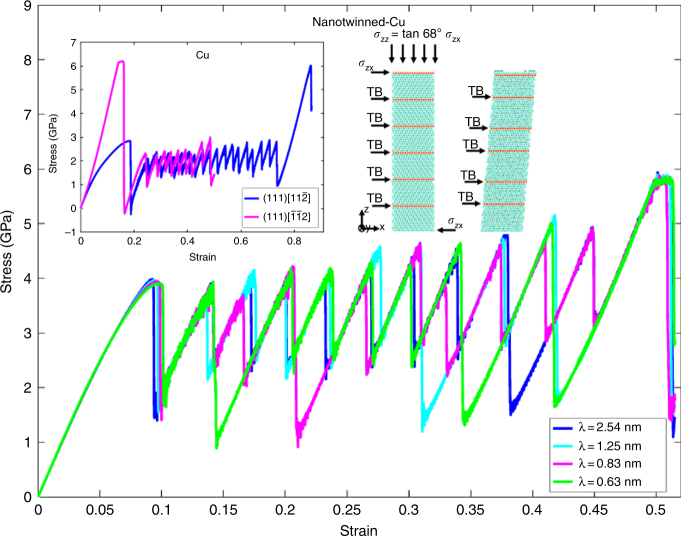
Stress-strain curves of single crystalline and nanotwinned-Cu, and simulation set-ups for molecular dynamics simulations on nanotwinned samples. Simulated stress-strain relations of nanotwinned-Cu samples with twin thickness varying from *λ* = 0.63 nm to 2.54 nm. The strength of the material and the tangent of the strain stiffening part are the same for samples with different twin boundary (TB) spacings. The simulation set-ups are shown in the right inset of the figure. The nantwinned-Cu super-cell is subjected to both shear and compressive loading similar to the Vickers indentation experiments. The compression to shear ratio is fixed at a constant value of tan (68°). The left inset presents the stress-strain curves for a shear loading of a single crystalline Cu along hard (111)$[\bar 1\bar 12]$ and weak (111)$[11\bar 2]$ shear directions. Different slopes of the curves confirm the anisotropic behavior of Cu crystal similar to covalent-bonding materials

Next, similar MD simulations were performed on nt-Cu samples, with the same dimensions of 40 × 21 × 50 nm^3^, containing TBs with thicknesses *λ* = 0.63 nm, 0.83 nm, 1.25 nm, and 2.54 nm. After 200 ps relaxation, the samples were subjected to both shear and compression loading with ratio fixed at a constant value of tan (68°), which is identical to the loading method applied in the original paper by Li et al.^1,2^ (see the right inset of Fig. 1). The simulated stress-strain curves in Fig. 1 for nt-Cu exhibit peak strengths which are insensitive to the TB spacing. The stress drops on the curves are caused by the movement of partial dislocations on TBs leading to TB migrations. The saw-tooth pattern continues until the nt-samples become twin-free and the strain stiffening takes place. The slope of the strain stiffening part of the curves is equivalent for all the samples as they share an identical twin-free single crystalline structure. It is noteworthy that the model in Fig. 1, which is identical to that used in refs. ^1^ and ^2^, cannot capture the TB-thickness dependence of strength as no grain boundaries have been considered.

The above simulations indicate that the strain stiffening mechanism proposed by Li et al.^1,2^ is not limited to nt-covalent bonding ceramics and exists also in nt-metals. Since it is known that nt-Cu exhibits softening behavior below a critical TB spacing^5,6^, this finding places significant doubt on the validity of the model of Li et al.^1,2^ in explaining the observed stiffening behaviors of nt-CBN and nt-Diamond.

## Data availability

All data generated and analyzed during this study are included in this published article.
